# A 15-Gene-Based Risk Signature for Predicting Overall Survival in SCLC Patients Who Have Undergone Surgical Resection

**DOI:** 10.3390/cancers15215219

**Published:** 2023-10-30

**Authors:** Sevcan Atay

**Affiliations:** Department of Medical Biochemistry, Faculty of Medicine, Ege University, 35100 Izmir, Turkey; sevcan.atay@ege.edu.tr

**Keywords:** small cell lung cancer, SCLC, transcriptomics, biomarker, prognosis, gene expression, risk signature, mRNA, surgery, resection

## Abstract

**Simple Summary:**

Small cell lung cancer (SCLC) is a high-grade neuroendocrine carcinoma with a poor prognosis, accounting for approximately 15% of lung cancer cases. Acquired resistance to standard chemotherapy is quite common in SCLC patients, and the survival benefit of surgery and the selection of surgical candidates are still controversial. This highlights the necessity of identifying predictive biomarkers that can serve to select patients who will benefit from various treatments and discovering novel molecular targets for SCLC treatment. In this study, for the first time, the association between tumoral transcriptional changes and prognosis was examined, and a novel multigene prognostic risk signature with strong discriminatory power was constructed and validated to predict the overall survival of SCLC patients who have undergone curative-intent surgical resection. The risk signature worked better than existing clinical and demographic parameters in predicting overall survival in patients with resected SCLC. Prognostic genes were predicted to have roles in pathways including regulation of transcription, cell cycle, cell metabolism, and angiogenesis.

**Abstract:**

Small cell lung cancer (SCLC) is a malignancy with a poor prognosis whose treatment has not progressed for decades. The survival benefit of surgery and the selection of surgical candidates are still controversial in SCLC. This study is the first report to identify transcriptomic alterations associated with prognosis and propose a gene expression-based risk signature that can be used to predict overall survival (OS) in SCLC patients who have undergone potentially curative surgery. An integrative transcriptome analysis of three gene expression datasets (GSE30219, GSE43346, and GSE149507) revealed 1734 up-regulated and 2907 down-regulated genes. Cox-Mantel test, Cox regression, and Lasso regression analyses were used to identify genes to be included in the risk signature. EGAD00001001244 and GSE60052-cohorts were used for internal and external validation, respectively. Overall survival was significantly poorer in patients with high-risk scores compared to the low-risk group. The discriminatory performance of the risk signature was superior to other parameters. Multivariate analysis showed that the risk signature has the potential to be an independent predictor of prognosis. The prognostic genes were enriched in pathways including regulation of transcription, cell cycle, cell metabolism, and angiogenesis. Determining the roles of the identified prognostic genes in the pathogenesis of SCLC may contribute to the development of new treatment strategies. The risk signature needs to be validated in a larger cohort of patients to test its usefulness in clinical decision-making.

## 1. Introduction

Small cell lung cancer (SCLC) is a high-grade neuroendocrine carcinoma with an abysmal prognosis, accounting for approximately 15% of lung cancer cases [1]. The lack of an effective therapeutic strategy for small cell lung cancer [2] highlights the absolute need to expand our knowledge of the molecular etiology of the disease to discover new drug targets.

High-throughput transcriptome profiling is one of the most utilized approaches to expand our understanding of the molecular mechanisms underlying diseases and discover novel biomarkers and therapeutic targets [3,4]. However, compared to other solid tumors, little is known about the clinical significance of the transcriptomic profile of SCLC due to limited tissues available for molecular studies, as curative surgical resection is not a major treatment modality for SCLC [5,6], and fine-needle aspirates are generally poor in quality and quantity [7,8,9,10]. As a solution to the small sample size that could result in low statistical power and consistency in transcriptomic analyses, integrative analyses of publicly available transcriptomic data have been carried out over the past five years, allowing the integration of results from various studies. However, in these studies, methodological approaches were used in which the effects of histological differences or different platforms on transcriptome profiles could not be excluded [11,12]. Other studies have reported data from an array capable of screening fewer than 10,000 genes in less than ten tumor samples, which may limit results and reduce reliability [13,14].

With the results of the study conducted by Cai et al. in 2021 [15], the publicly available cumulative transcriptomic data of SCLC has reached a level that allows integrative analysis of transcriptomic data obtained from the same microarray platform. In this study, to increase reliability by eliminating the potential effects of array platform and data processing on transcriptome profiles, microarray data from a single platform was included in the analysis, and all signal intensities were subjected to the same normalization process. In addition, only transcriptomic data from human primary SCLC tissues collected by surgical resection were included to ensure homogeneity in the study group. Thus, transcriptomic changes in SCLC tumor tissues compared to non-tumoral lung tissues were identified by integrative transcriptome analysis. This is the first study to investigate associations between all gene expression changes identified by whole transcriptome data analysis and overall survival of SCLC patients.

The presented study aims to provide insight into the molecular mechanisms associated with prognosis, propose new therapeutic targets, and suggest a novel risk signature that may serve as a promising prognostic indicator for the overall survival of SCLC patients who have undergone curative-intent surgical resection.

## 2. Materials & Methods

### 2.1. Selection of Microarray Datasets

NCBI Gene Expression Omnibus (http://www.ncbi.nlm.nih.gov/geo/ (accessed on 1 April 2022)) was systematically searched for eligible datasets using the keywords ‘small cell lung cancer’. The inclusion criteria were: (*i*) gene expression microarray data, (*ii*) human-derived primary small cell lung cancer tissues, matched adjacent non-tumor tissue samples or non-tumor lung tissues, and (*iii*) tumor tissues obtained by curative-intent surgical resection.

### 2.2. Integrative Transcriptome Analysis

CEL. files of eligible datasets were downloaded and imported to PARTEK Genomics Suite version 7.0 (PGS; Partek, St. Louis, MO, USA). The raw probe intensities were RMA (Robust Multichip Average) normalized and log2 transformed. A two-way analysis of variance (ANOVA) was performed on the data using the study name as a variable, and batch effects of the studies were removed. Partial least squares (PCA) and hierarchical clustering analyses were used to identify outliers and variables with significant effects on the data. The differentially expressed genes (DEGs) were identified by following the gene expression workflow within PGS. ANOVA generated a list of genes significantly different between SCLC and control tissues (|log2FC| ≥ 1 and FDR adj. *p* < 0.05).

### 2.3. Gene Ontology and Pathway Enrichment Analysis of the DEGs

Pathway enrichment analysis (GSEA) and Gene Ontology analysis were performed to predict the dysregulated pathways underlying disease and generate insights into the biological processes, molecular functions, and cellular locations affected in SCLC. Analyzes were performed using the WEB-based GEne SeT AnaLysis Toolkit 2019 (WebGestalt) with a cut-off criterion of FDR ≤ 0.05 [16]. In GSEA, the enrichment category was selected by the Kyoto Encyclopedia for Genes and Genomes (KEGG) [17]. The category size was calculated based on the number of overlapping genes between the annotated genes in the category and the ranked gene list for the GSEA method. Categories smaller than five and greater than 2000 were removed during the analysis. In the Gene Ontology analysis, enriched gene clusters were post-processed by affinity propagation method using R packet apcluster to reduce redundancy.

### 2.4. Protein-Protein Interaction Network Analysis

PPI Network Analysis evaluated interactions between proteins encoded by the identified up-and down-regulated genes. Protein-protein interactions with a confidence score greater than 0.9 calculated by the Search Tool for the Retrieval of Interacting Genes (STRING, version 11.5) were used to visualize and analyze the PPI network by Cytoscape Software (version 3.9.1) [18,19]. 

Hub proteins predicted to play a critical role in the organization of the PPI network and thus, potentially in the pathogenesis of SCLC were identified by NetworkAnalyzer, a CytoScape tool, by detecting nodes with high edge counts. Nodes with a degree (number of edges connected to the node) ≥40 were considered to represent ‘hub proteins’.

The highly connected regions (clusters) in the constructed networks were identified by MCODE, a Cytoscape App, with the parameters false degree cutoff 2 and K-Core 2 [20]. Since clusters in a PPI network often indicate protein complexes or parts of pathways, Gene Ontology biological process categories of the clusters were identified by STRING PPI Enrichment analysis. The most significant GO Biological process category was reported for proteins in each cluster analyzed (*p* ≤ 0.05).

### 2.5. Finding Prognostic DEGs for SCLC

The prognostic values of the identified up-and down-regulated genes in SCLC were evaluated in the small cell lung cancer dataset (U. Cologne, Nature 2015 Dataset in www.cbioportal.org/ (accessed on 1 July 2022)), (Dataset ID: EGAD00001001244, Platform: Illumina HiSeq 2000). This dataset is the largest SCLC cohort reporting transcriptomic data with overall survival [21]. The dataset initially includes log2 FPKM values from 81 human primary SCLC tumor specimens and clinical data of the patients. 

The identified prognostic gene signature was validated in an independent dataset, GSE60052 (Platform: Illumina HiSeq 2000, *n* = 48). The dataset includes transcriptomic data from FFPE archived SCLC tissue samples collected by surgical resection. It has been reported that samples were subjected to macro-dissection, and tumor purity was >70% [22]. 

From these datasets, only transcriptomic data from tumor tissues meeting the following criteria were included in the further analysis: (*i*) the tumor sampling procedure is surgical resection, (*ii*) the sample type is primary SCLC, and (*iii*) patients with more than 2 months of follow-up.

The Kaplan Meier Plotter (http://kmplot.com/analysis/ (accessed on 7 July 2022)) [23], a web-based survival analysis tool that allows researchers to perform survival analysis and generate Kaplan-Meier survival plots using custom-generated data, was used to assess the correlation between FPKM values of the identified DEGs and overall survival in patients with SCLC from EGAD00001001244 dataset. The patient cohort was dichotomized into two groups using the auto-select best cut-off option in the Km Plotter. The significance is computed using the Cox-Mantel (log-rank) test, and the false discovery rate (FDR) is computed using the Benjamini–Hochberg method. A log-rank *p*-value ≤ 0.05 (FDR ≤ 0.05) was considered statistically significant. Then differentially expressed genes, which were found to be significantly associated with overall survival in patients, were further evaluated with Cox’s proportional hazards regression analysis using IBM SPSS Statistics for Windows (v.28.0.1.1.(15), IBM corp.: Armonk, NY, USA), (*p*-value ≤ 0.05). To prevent overfitting, least absolute shrinkage, and selection operator (LASSO) regression analysis was conducted on the identified candidate prognostic genes using the R package “glmnet” in XLSTAT, and the optimal penalty parameter λ was determined by 10-fold cross-validation [24]. The risk score was constructed by the linear combination of each individual normalized expression value of the identified predictive genes weighted by their corresponding Lasso regression coefficients to avoid the problem of overfitting [25]. The risk score for each patient was calculated according to the following formula:

$$RS=\underset{i=1}{\sum }Coefficient\;\left(mRNAi\right)XExpression\;\left(mRNAi\right)$$

### 2.6. Internal and External Validation of the Risk Score

For internal validation, the EGAD00001001244 dataset was randomly divided into a training cohort (60%, *n* = 44, event(*n*) = 23) and a validation cohort (40%, *n* = 30, event(*n*) = 23) using XLSTAT data sampling (Seed (random numbers): 4629237). GSE60052 dataset (*n* = 45, event(*n*) = 21) was used for external validation. The cohorts were stratified into high- and low-risk groups according to the median risk score. Kaplan–Meier survival analysis was used to evaluate the effect of risk score on overall survival. A Cox–Mantel (log-rank) *p*-value ≤ 0.05 was accepted as significant. Univariate and multivariate Cox proportional hazards regression analyses were performed to identify individual factors associated with overall survival and to assess whether the risk score was an independent predictor of prognosis, respectively (IBM SPSS Statistics for Windows (v.28.0.1.1.(15), IBM corp.: Armonk, NY, USA). The diagnostic efficacy of the risk model was assessed by receiver operating characteristic (ROC) curves and the area under the curve (AUC). The differential expression of the genes included in the risk score was evaluated in five datasets (two internal and three external datasets: GSE30219 [26], GSE40275 [27], GSE149507 [15], GSE60052 [22] and GSE108055 [28]). GEO2R was used to identify genes differentially expressed in SCLC tumors compared to normal lung tissues [29]. GSE108055 was analyzed using NetworkAnalyst (https://www.networkanalyst.ca/ (accessed on 21 September 2023)) [30]. It was considered significant if the false discovery rate was 0.05≥ and the change in gene expression was |log2FC| ≥ 1. Kaplan-Meier survival, violin plots, and ROC curves were generated using (GraphPad Prism v.8.0.1. for Windows, GraphPad Software, Boston, MA, USA). A *p*-value ≤ 0.05 was considered statistically significant.

## 3. Results

**Figure 1:**
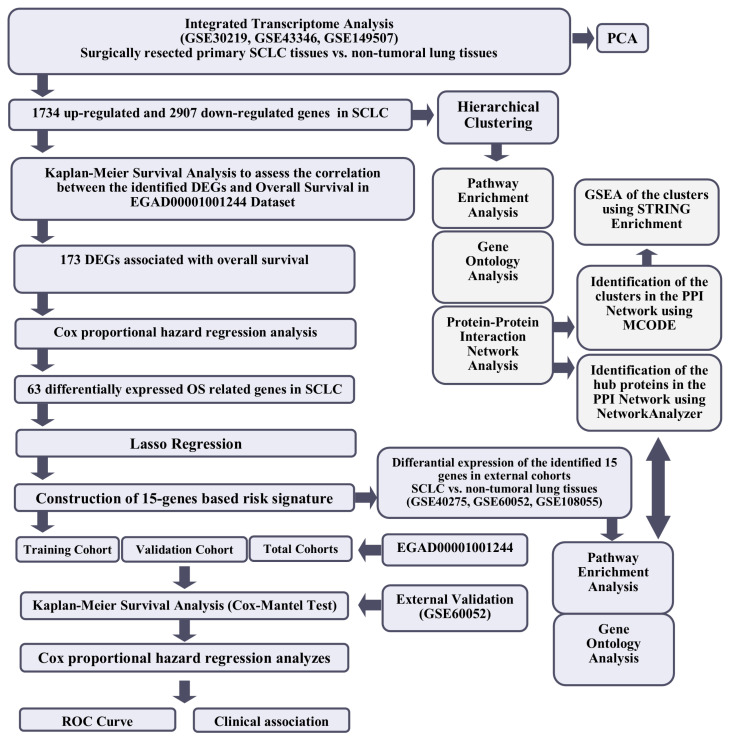
The workflow of the study.

The workflow of the study is shown in Figure 1.

### 3.1. Differentially Expressed Genes in SCLC

An integrative transcriptome analysis approach was used to identify the up-and down-regulated genes in primary small cell lung carcinoma tissues compared to adjacent non-tumoral and normal lung tissues. Three datasets (GSE30219 [26], GSE43346 [31] and GSE149507 [15]), including transcriptomic data of a total of 62 primary SCLC tissues and 32 control lung tissues, from the Affymetrix Human Genome U133 Plus 2.0 Array platform were included in the analysis (Table 1). 

**Figure 2:**
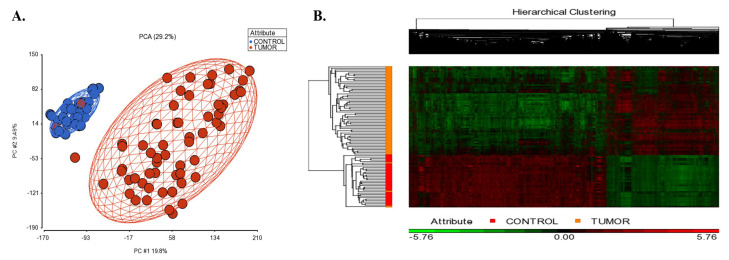
(**A**) 2D scatter plot of principal component analysis (PCA) results of microarray data included in the integrative transcriptome analysis. Controls are shown in blue color, and the small cell lung cancer group is colored in red. Each circle represents an individual sample’s global gene expression profile, and samples with similar profiles are clustered together. (**B**) Hierarchical clustering of the identified DEGs in SCLC. The signal intensities were RMA normalized and transformed to the log2 scale. Red and green colors indicate differentially up and down-regulated genes, respectively.

Figure 2A shows the PCA scatterplot revealing that tissue type (tumor vs control tissues) is the largest source of variability in the data. The gene expression analysis in PGS detected 1734 up-regulated and 2907 down-regulated unique genes in SCLC tissues compared to controls (|log2FC| ≥ 2, and FDR adj. *p* < 0.05; Supplementary File S1A). The hierarchical clustering of the differentially expressed genes is shown in Figure 2B. 

INSM1 (insulinoma-associated 1), a zinc-finger transcription factor implicated as a driver of neuroendocrine differentiation in multiple tissues [32], was the top over-expressed gene in SCLC (FC = 71.8814, FDR adj. *p* = 2.23 × 10^−16^). SFTPA1///SFTPA2 (surfactant protein A1///surfactant protein A2) showed the most significant down-regulation in SCLC compared to control tissues (log2FC = −128.699, FDR adj. *p* = 1.26 × 10^−20^). 

### 3.2. Construction of a Tumor-Based Prognostic Risk Signature

Among the DEGs identified in the SCLC integrative-analysis cohort, expression data for 1579 up-regulated genes and 2815 down-regulated genes were available in the EGAD00001001244 Dataset (*n* = 74). The relationship between the mRNA expression of these available genes and overall survival was questioned using the Cox–Mantel (log-rank) test. The mRNA expression levels of 173 genes (3.93%) were found to be associated with the overall survival in patients with SCLC (FDR ≤ 0.05 and log-rank *p* value < 0.05). The output of the 173 gene is shown in Supplementary File S1B. 

First, univariate survival analyses were performed using the Cox proportional hazards regression methodology to identify individual risk factors related to overall survival (*p* value < 0.05). By Cox regression analysis, among the identified 173 candidate prognostic genes, 69 (34 and 35 genes identified as up-regulated and down-regulated in SCLC, respectively) retained significant associations with overall survival. Five genes that violated the proportional risk assumption were eliminated (*p* < 0.05). The univariate Cox regression analysis results for overall survival are listed in Supplementary File S1C.

**Figure 3:**
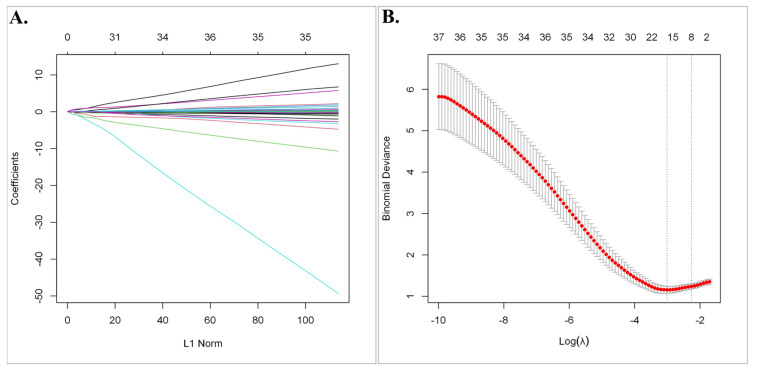
The least absolute shrinkage and selection operator (LASSO) regression analysis. (**A**) The Lasso coefficient profile plot of the 63 candidate prognostic genes in the EGAD00001001244 Dataset was constructed against the log (lambda) sequence. (**B**) Selection of optimal penalty parameter (λ) according to 10-fold cross-validation.

**Figure 4:**
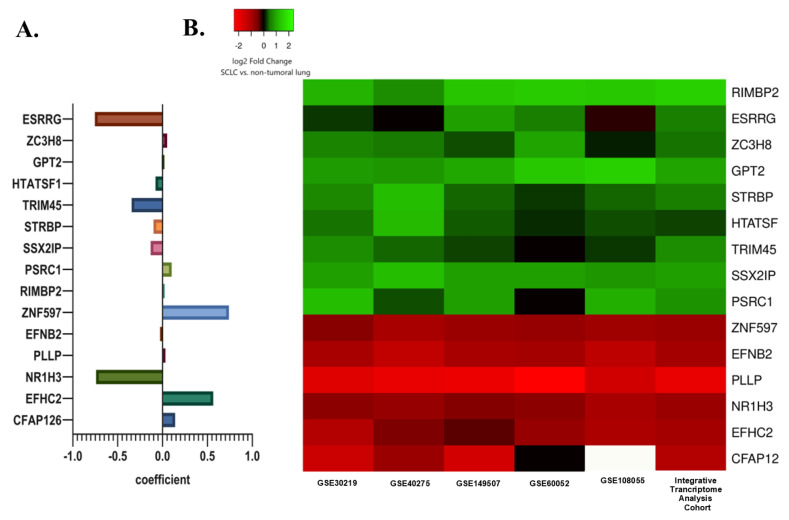
(**A**) The selected prognostic genes obtained from the LASSO regression model and their risk coefficients. The exact values can be seen in the constructed risk formula. (**B**) A heat map showing the differential mRNA expression of selected prognostic genes in SCLC tumor tissues relative to non-tumoral lung tissues in the internal and external cohorts. The green color indicates up-regulation, the red color indicates down-regulation, and the white color indicates missing data.

Subsequently, least absolute shrinkage and selection operator (LASSO) regression analysis was utilized to select the gene set with the optimized predictive ability associated with overall survival in SCLC cohort (lambda = 0.049229, alpha = 1) (Figure 3A,B). A total of 15 genes selected by the algorithm were used to develop the risk score. The genes included in the risk score and corresponding coefficients are shown in Figure 4A. 

In the integrative transcriptome analysis cohort, ESRRG, ZC3H8, RIMBP2, GPT2, STRBP, HTATSF1, TRIM45, SSX2IP, and PSRC1 were up-regulated while ZNF597, EFNB2, PLLP, NR1H3, EFHC2, and CFAP126 were down-regulated genes in SCLC tumor tissues compared to non-tumoral lung tissues. 

A very similar differential gene expression pattern in three external SCLC datasets (GSE40275, GSE60052, and GSE108055) was shown by a heat map. Differential expression levels were also evaluated in two datasets from the integrative transcriptome analysis cohort, including a control group (GSE30219 and GSE149507) to demonstrate whether the detected difference was due to a single dataset (Figure 4B).

**Figure 5:**
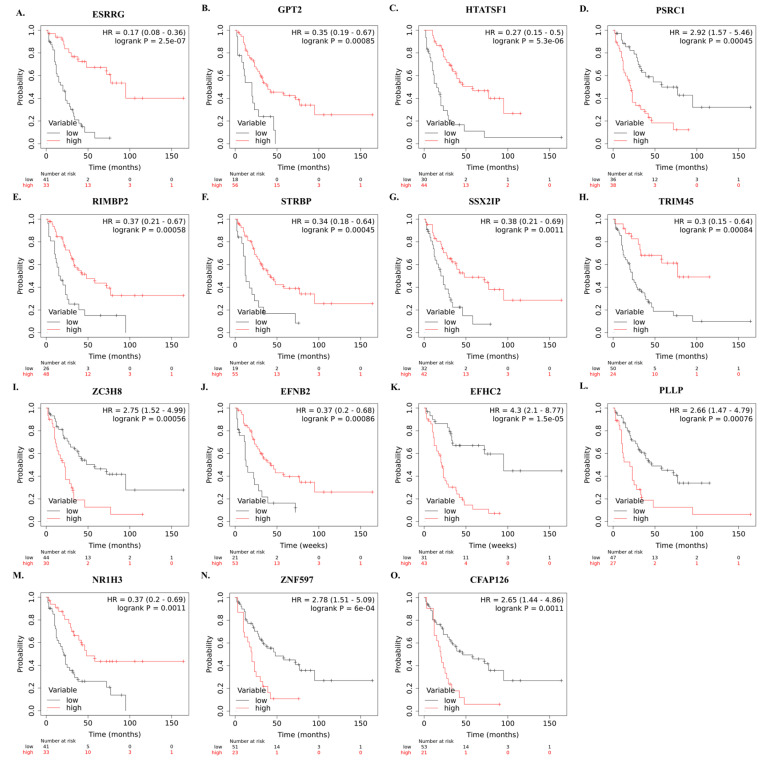
Kaplan–Meier survival curves showing the correlation between identified high and low mRNA levels of the 15 genes and overall survival in SCLC patients after curative intent surgical resection (**A**–**O**). Survival plots were created using Km-Plotter using EGAD00001001244 data.

The correlation between mRNA expression of selected genes and overall survival of SCLC patients who underwent curative-intent surgical resection is shown in Figure 5A–O.

The risk score was calculated for each patient as follows: 

Risk Score = (ESRRG × (−0.75493)) + (HTATSF1 × (−0.07884)) + (PSRC1 × (0.101006)) + (RIMBP2 × (−0.00743)) + (STRBP × (−0.10136)) + (SSX2IP × (−0.13313)) + (TRIM45 × (−0.34483)) + (ZC2H8 × (0.050896)) + (GPT2 × (−0.00902)) + (EFNB2 × (−0.02784)) + (EFHC2 × (0.564705)) + (PLLP × (0.032548)) + (NR1H3 × (−0.74115)) + (ZNF597 × (0.739434)) + (CFAP126 × (0.139897)).

### 3.3. Validation of the Risk Signature

**Figure 6:**
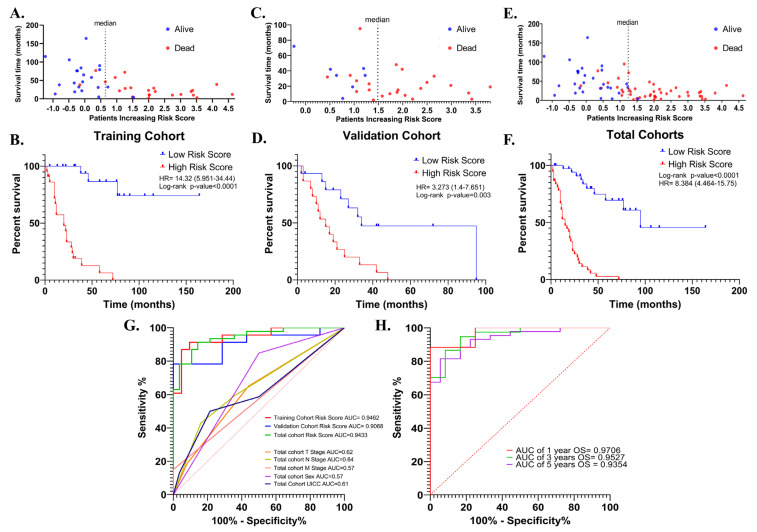
The distribution of the risk scores in the training, validation, and total cohorts ((**A**,**C**) and (**E**), respectively). Kaplan–Meier survival curve for OS of high-and low-risk SCLC groups in the training, validation, and total cohorts ((**B**,**D**) and (**F**), respectively). ROC curves of the risk score for OS in the training, validation, and total cohorts. ROC curves of T-, N-, M- stages, UICC stage, and sex for OS in the total cohort were shown (**G**). ROC curves for 1-, 3-, and 5-year OS in the total cohort based on risk signature (**H**).

The risk signature was first evaluated with Kaplan–Meier survival analysis (Cox–Mantel test) for the potential association between high- and low-risk scores and overall survival in training (*n* = 44), validation (*n* = 30) and total cohorts (*n* = 74). In all cohorts, patients were divided into high- and low-risk groups according to median risk score. In the training cohort, a high-risk score was shown to be associated with reduced overall survival (HR = 14.32 (5.951–34.44), log-rank *p* < 0.0001) (Figure 6A,B). Similar results were obtained in the validation cohort (HR = 3.27 (1.4–7.65) log-rank *p* = 0.003) (Figure 6C,D), and total cohort (HR = 8.38 (4.46–15.75), log-rank *p* < 0.0001) (Figure 6E,F). 

Then, the discriminative performance of the risk signature was evaluated by ROC analysis (receiver-operating characteristic analysis). The AUCs with 95% Cls of ROC curves for predicting overall survival were 0.94 (0.88–1.0), 0.90 (0.79–1.0) and 0.94 (0.89–0.99) in training, validation, and total cohorts, respectively (Figure 6G). The ROC curves showed good discrimination for the 1-, 3- and 5-year OS AUCs of 1-, 3- and 5-year survival of SCLC in the total cohort were 0.97 (0.899–1), 0.95 (0.893–1), and 0.9354 (0.87–0.99), (Figure 6H). These results showed that the risk signature works as a very good measure of separability. 

Then, the effects of the risk score and the patients’ demographic and clinical characteristics on overall survival were evaluated with univariate and multivariate Cox proportional hazards regression analysis. The clinical and demographic characteristics of the patients from the EGAD00001001244 cohort and the results of the analyses are listed in Table 2.

In the training cohort, overall survival was lower in UICC III + IV patients compared to UICC I + II patients (HR = 2.29 (0.99–5.27); *p* = 0.05). Being female was significantly associated with longer overall survival in both the validation (HR = 0.32 (0.14–0.73); *p* = 0.007) and total cohorts (HR = 0.06 (0–0.4); *p* = 0.007). In addition, radiotherapy, chemotherapy, and age were not associated with survival in all three cohorts (*p* > 0.05). The effect of neoadjuvant therapy on overall survival could not be evaluated, as most of the tumor samples selected for the analysis were treatment naïve (*n* = 72, 97.29%), but this also minimized the potential impact of therapeutics on transcriptomic data from tumors. The risk score was associated with worse overall survival in the training (HR = 2.08 (1.59–2.73); *p* < 0.0001) validation (HR = 2.01 (1.28–3.15); *p* = 0.002), and total cohorts (HR = 2.07 (1.67–2.55); *p* < 0.0001). The fact that the risk score is the only parameter that maintains its association with worse overall survival in all three groups indicates that the risk score has the potential to be a more robust measure than other parameters in predicting overall survival (Table 2). Indeed, when the AUC values were compared, the discriminatory power of the risk score was superior to the others (Figure 6G).

The parameters associated with overall survival in univariate Cox regression analysis were then used in multivariate Cox regression analysis to question whether the risk score was an independent predictor for overall survival. In multivariate analysis, the risk score retained a significant association with overall survival in training (HR = 2.12 (1.59–2.82), *p* < 0.0001), validation (HR = 1.66 (1.02–2.69), *p* = 0.04), and total cohorts (HR = 2.04 (1.64–2.54), *p* < 0.0001). 

**Figure 7:**
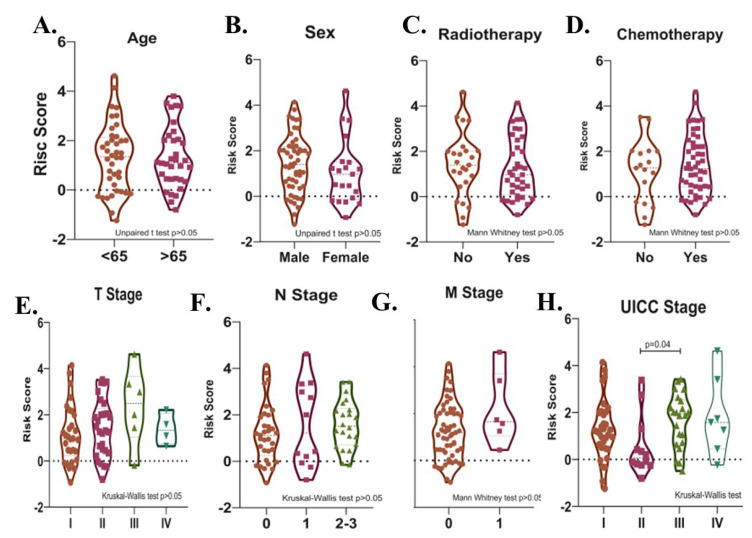
Violin plots show the relationships between risk score levels and age (**A**), sex (**B**), radiotherapy (**C**), chemotherapy (**D**), T- (**E**), N- (**F**), and M- stage (**G**), and UICC stage (**H**) in SCLC patients.

**Figure 8:**
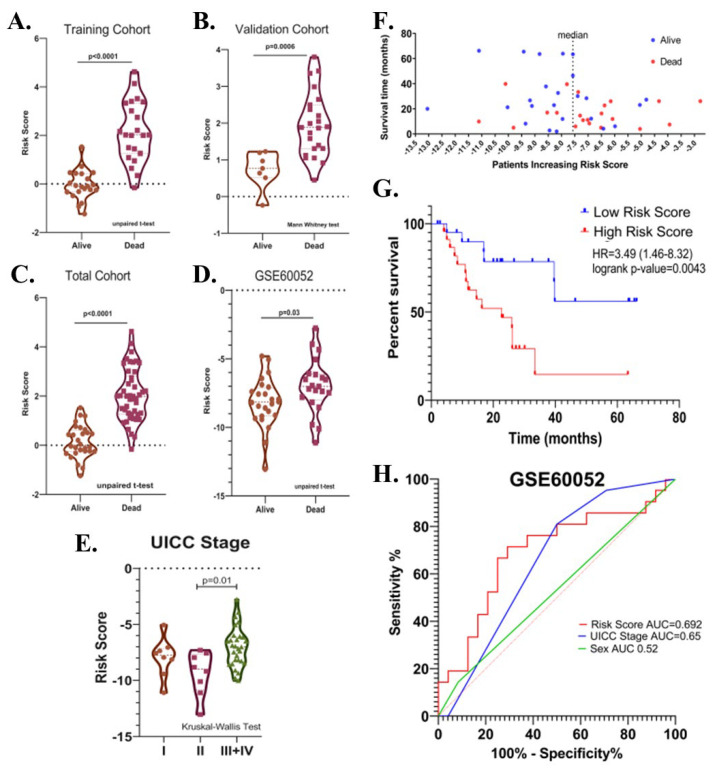
(**A**) Violin plot graphs show risk score levels in deceased and surviving patients in training (**B**), validation (**C**), total, and (**D**) test (GSE60052) cohorts. (**E**) The violin plot shows the relationship between risk score and the UICC stage. (**F**) The distribution of the risk scores in the test cohort. (**G**) Kaplan-Meier survival curve for OS of high-and low-risk SCLC groups in the test cohort. (**H**) ROC curve of sex, UICC- stage, risk score and their respective AUC values in the test cohort.

Potential relationships between the risk score and other parameters were then evaluated. There was no significant difference in terms of risk score between male and female patients, between patients with different T-, N-, M- stages, between patients over and under 65 years of age, and between patients who received and did not receive radiotherapy or chemotherapy after surgery in the total cohort (*p* > 0.05). (Figure 7A–G). The risk score was higher in UICC stage III than in Stage II (*p* = 0.04, Figure 7H). Risk score was found to be higher in deceased patients compared to patients who survived in all three cohorts (*p* < 0.05, Figure 8A–C).

The prognostic significance of the identified risk score in SCLC was further evaluated in the GSE60052 dataset (*n* = 45, event(*n*) = 21). The GSE60052 dataset contains transcriptomic data (platform: Illumina HiSeq 2000) from surgically resected primary SCLC tissues. Unlike patients in the EGAD00001001244 dataset, all patients in the GSE60052 dataset were reported to have received at least one cycle of chemotherapy after surgery. In addition, the proportion of treatment naïve samples was 84.44%. Patients were divided into two groups according to the median risk value. Kaplan–Meier survival analysis showed that a high-risk score was associated with decreased overall survival (Cox–Mantel test, HR = 3.49 (1.46–8.32), *p* = 0.0043) (Figure 8F,G). Among covariates, including risk score, UICC stages, sex, age, and chemotherapy, only UICC-stage (HR = 7.34 (2.04–26.34), *p* = 0.002) and risk score (HR = 3.84 (1.43–10.33), *p* = 0.008) were found to be associated with the overall survival by univariate Cox regression analysis. In multivariate analysis, UICC-stage and risk score were associated with overall survival in patients (high-risk score; HR = 2.96 (1.03–8.45), *p* = 0.04; UICC-stage HR = 6.18 (1.63–23.34), *p* = 0.007). The predictive value of the risk score, sex, and UICC stage was evaluated by area under the curve (AUC) scores for overall survival produced by receiver operating characteristic (ROC) analysis. Although the discriminatory power of the risk score was relatively poor in GSE60052 (AUC = 0.6925 (0.53–0.85), it was better at predicting overall survival than sex and UICC-stage (AUCs (%95Cl); 0.52 (0.35–0.70) and 0.65 (0.49–0.81), respectively), (Figure 8H), consistent with the previous result (Figure 6G). As in the EGAD00001001244 cohort, the risk score was higher in deceased patients than in surviving patients (*p* = 0.03) (Figure 8D), and in UICC stage III + IV than in UICC stage II (*p* = 0.01) (Figure 8E).

### 3.4. Functional Enrichment and Protein–Protein Interaction Analysis

The 15 genes in the risk signature were not significantly enriched in any gene ontology category or pathway (FDR > 0.05). There was no evidence in the PPI network analysis that these 15 genes directly interact with each other. To determine the pathways in which the identified prognostic genes function in the SCLC pathological molecular mechanism and the proteins they interact with, functional enrichment and PPI network analyses were first performed using the DEGs obtained from the integrative transcriptome analysis. In the PPI network of identified DEGs, subnetworks were created containing the proteins encoded by the identified prognostic genes and their first neighbors. Then, functional analyses were performed for the proteins involved in these sub-PPI interaction networks. Functional enrichment analysis results obtained from subnetworks were compared with those of analyses using DEGs; thus, the positions and functions of prognostic genes within the SCLC pathological mechanism were predicted.

### 3.5. Gene Ontology and Pathway Enrichment Analyzes of the DEGs

Gene Ontology and Pathway Enrichment analyses (GSEA) of the DEGs were performed to identify pathways, biological processes, molecular functions, and cellular locations impacted in SCLC. 

**Figure 9:**
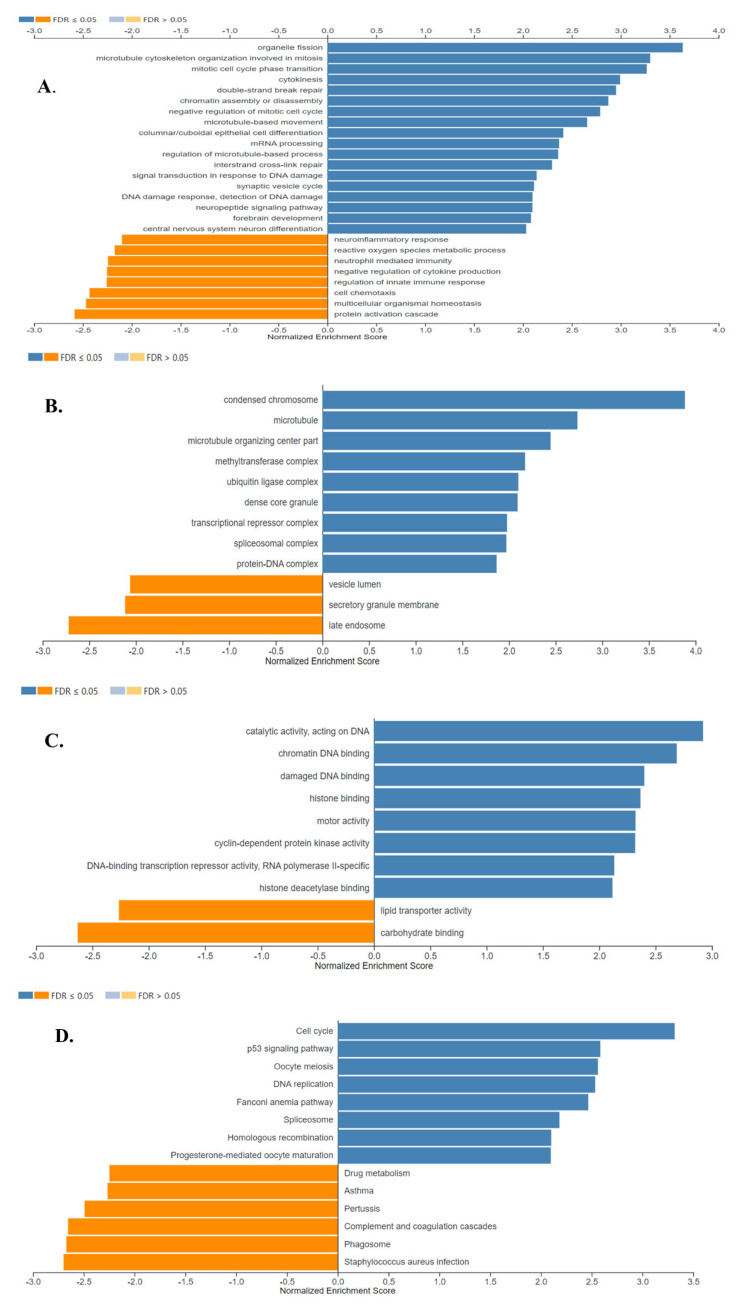
Gene Ontology Analysis and Gene Set Enrichment Analysis of the differentially expressed genes in SCLC. Enriched biological processes (**A**), cellular locations (**B**), and molecular functions (**C**) associated with the differential gene expression in SCLC were shown. (**D**) The identified enriched KEGG pathways in SCLC. Enriched positively and negatively related categories are shown in blue and orange color, respectively (FDR ≤ 0.05). Analyses were performed using WebGestalt 2019.

The results of the gene ontology analysis correlated with the results of the GSEA analysis, showing that the detected up-regulated genes were associated with cell division, transcriptional and epigenetic regulation, and DNA repair, while down-regulated genes were found to be associated with secretion -which was mostly represented by the down-regulation of genes encoding surfactant proteins-, and immune response proteins. The results of gene ontology biological process, cell component, and molecular function analyses are shown in Figure 9A, B and C, respectively (FDR ≤ 0.05).

Figure 9D shows the eight positively and six negatively associated enriched KEGG pathways identified in SCLC (FDR ≤ 0.05). Enriched positively related pathways included Cell cycle, p53 signaling pathway, DNA replication, DNA repair, and transcriptional regulation. Proteins encoded by the down-regulated genes were major tissue compatibility complex class II proteins, proteins involved in the complement and coagulation cascades and drug metabolism (FDR ≤ 0.05). 

### 3.6. Protein–Protein Interaction (PPI) Network Analysis of the DEGs

**Figure 10:**
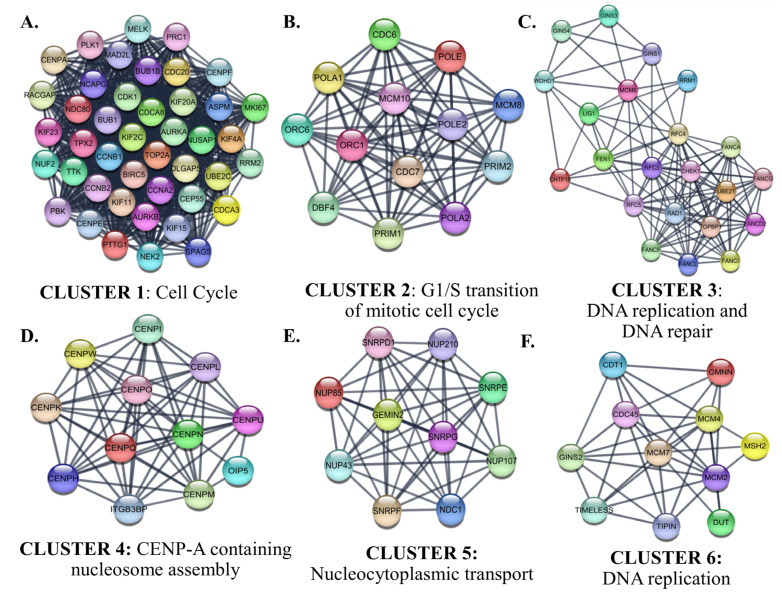
The identified clusters in the protein-protein-interaction network of the up-regulated genes in SCLC. The protein-protein interaction (PPI) networks were constructed by Cytoscape based on the PPI correlations from the STRING database. The clusters in the networks were identified using MCODE. Six clusters with an MCODE score > 5 and the results of the gene ontology biological process analysis of each cluster are shown (**A**–**F**). Functional analyses were performed using STRING Enrichment.

Interactions between proteins encoded by the identified up-and down-regulated genes were evaluated by PPI Network Analysis. The identified hub proteins in the PPI network are listed in Supplementary File S1D. The identified clusters in the protein-protein-interaction network of the up-regulated genes in SCLC are shown in Figure 10.

Cluster 1 for the identified up-regulated genes was enriched in the ‘*mitotic cell cycle*’ category. CDK1, which has the highest degree among the hub proteins, was also included in Cluster 1. The biological processes of the proteins in Clusters 2, 3, and 6 were intertwined and closely related. Cluster 2 contained proteins involved in the processes of ‘*G1/S transition of mitotic cell cycle*’. CDC6, which participates in the initiation of DNA replication and checkpoint controls that ensure DNA replication is completed before mitosis initiates, was the highest-ranked hub protein in Cluster 2. Cluster 2 also contained subunits of origin recognition complex (ORC6/1) that binds origins of replication, polymerases, primases, and helicase (MCM complex), which are essential components for DNA replication and homologous recombination DNA repair. The top-ranked hub protein included in Cluster 3 was CHEK1 (*Checkpoint Kinase 1*). CHEK1 is a serine/threonine-protein kinase required for checkpoint-mediated cell cycle arrest and activation of DNA repair in response to the presence of DNA damage or unreplicated DNA. The cluster also includes other proteins that have roles in DNA repair and replication, such as TIMELESS, which is required to stabilize replication forks during DNA replication by forming a complex with TIPIN. This complex regulates DNA replication processes under both normal and stress conditions and influences both CHEK1 phosphorylation and the intra-S phase checkpoint in response to genotoxic stress. While Cluster 4 included centromere complex proteins, which are involved in the assembly of kinetochore proteins, mitotic progression, and chromosome segregation, Cluster 5 included spliceosomal small nuclear ribonucleoproteins (snRNPs), which are the building blocks of the spliceosome, and nucleoporin family members. 

**Figure 11:**
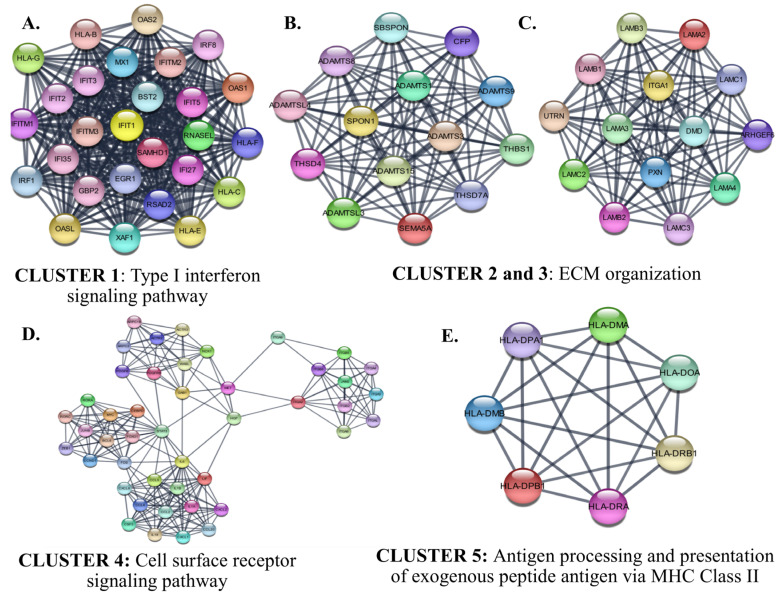
The identified clusters in the protein-protein-interaction network of the down-regulated genes in SCLC. The protein–protein interaction (PPI) networks were constructed by Cytoscape based on the PPI correlations from the STRING database. The clusters in the networks were identified using MCODE. Five clusters with an MCODE score >5 and the results of the gene ontology biological process analysis of each cluster are shown (**A**–**E**). Functional analyses were performed using STRING Enrichment (*p* < 0.05).

The identified six protein–protein interaction clusters with the highest scores in the PPI network of the down-regulated genes in SCLC are shown in Figure 11. The identified clusters were associated with an immune response such as ‘*Type I interferon signaling*’, ‘*cytokine-mediated signaling associated cell surface receptor signaling pathway*’, and ‘*antigen processing and presentation of exogenous peptide antigen* via *MHC Class II*’. The hub proteins in the identified Clusters were STAT3, FOS, PXN, and ITGA1.

### 3.7. Functional Analysis of the Risk Signature

**Figure 12:**
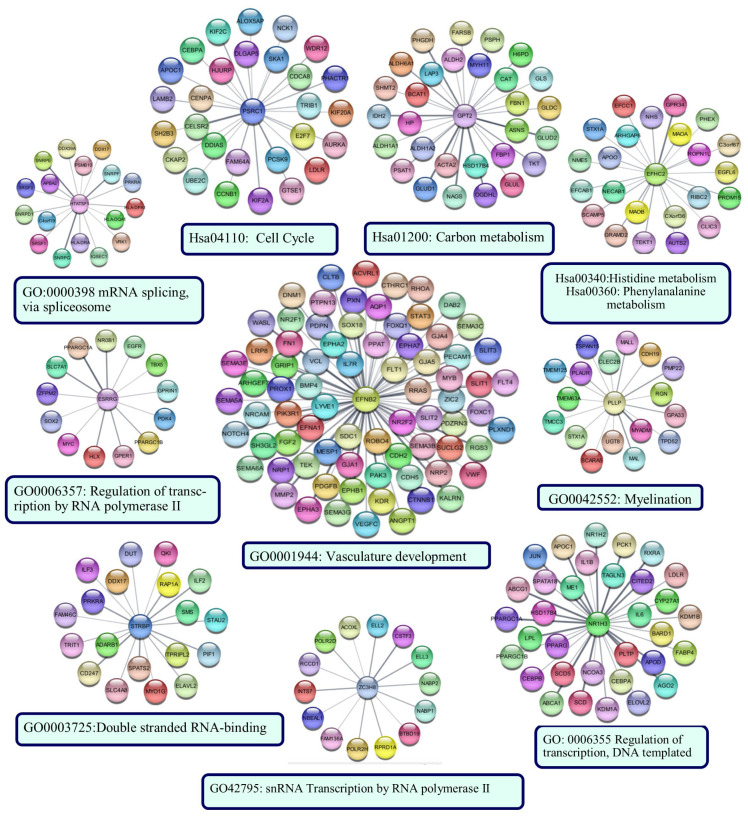
The sub-PPI networks formed by the proteins encoded by the genes in the risk signature and their first neighbors. Cytoscape constructed the protein −protein interaction (PPI) networks based on the PPI correlations from the STRING database. Functional analyses were performed using STRING Enrichment.

The sub-PPI networks formed by the proteins encoded by the genes in the risk signature and their first neighbors are shown in Figure 12. The results of the functional enrichment analysis performed for each sub-network are indicated below the networks (*p* < 0.05). 

The protein with the most edges in the PPI network was EFNB2. The EFNB2 network was enriched in the *‘vasculature development*’ category. The network included hub proteins PIK3R1, CTNNB1, STAT3, and FN1. The PSRC1 network was enriched in the ‘*Cell cycle*’ category, as was the PPI network’s largest cluster of up-regulated genes. Identified hub proteins, including CCNB1, AURKA, DLGAP5, UBE2C, and CENPA, were the first neighbors of PSRC1. NR1H3, HTATSF1, ESRRG, STRBP, and ZCH8 were enriched in categories generally associated with transcriptional regulation, RNA processing, and post-transcriptional regulation of gene expression. Hub proteins EGFR and JUN were in the networks of ESRRG and NR1H3, respectively. GPT2 and EFHC2 were enriched in categories related to cellular metabolism. PLLP network was associated with ‘*myelination*’. CFP126, TRIM45, RIMBP2, and SSX2IP did not form a sub-network. ZNF597 formed a sub-network with four edges: TMEM243, PRIM2, LIMF1, and L3MBTL1. However, they were not significantly enriched in any functional category. 

## 4. Discussion

Standard chemotherapy for SCLC consisting of platinum plus etoposide or anthracycline-based regimens has shown a high response rate; unfortunately, the development of acquired resistance in patients is quite common, and the recurrent disease is almost invariably lethal [33,34]. Unlike other solid tumors, there has been little progress in new treatment strategies and improving SCLC patients’ overall survival. Although current American Society of Clinical Oncology and European Society for Medical Oncology guidelines indicate that surgical resection should be considered only in patients with stage I SCLC [35,36], curative surgery may be preferred as part of comprehensive treatment in both early and advanced SCLC patients [5]. However, the survival benefit of surgery in SCLC is still controversial [37,38], and the selection of surgical candidates for SCLC is still arbitrary [39]. Therefore, there is a need to elucidate the molecular etiology of the disease to suggest more effective treatment strategies and to identify new diagnostic and predictive biomarkers that aid clinical decision-making. 

In the current study, using stringent inclusion and exclusion criteria examining patient selection, an integrative analysis of publicly available transcriptomic data was performed to provide new insights into the molecular mechanisms underlying the disease. Additionally, a novel mRNA expression-based risk signature that could predict overall survival in SCLC patients who underwent curative-intent surgical resection was developed and evaluated. 

The first step in creating the risk score was to identify genes that were expressed differently in SCLC compared to non-tumoral lung tissues by integrative transcriptomic data analysis. Transcriptomic data from a total of 62 primary SCLC tumor tissues and 32 non-tumor lung tissues were included in the study. This sample size has been reported to be sufficient to detect changes in 2-fold changes of expression level for the 90% least variable genes (FDR = 0.01, power = 0.9) [40]. Although adjacent non-tumor tissues are generally accepted as healthy controls, there are also studies reporting that, at the molecular level, these tissues are distinct from both healthy and tumor tissues and may represent an intermediate state [41,42]. PCA and hierarchical cluster analyses in this study showed that adjacent non-tumor and normal lung tissues had very similar signal intensities and transcriptomic profiles, supporting that they could represent the control group. INSM1 is a new neuroendocrine marker reported to be positive in the most common subtypes of SCLC [1,43,44,45]. Consistent with these data, in this study, INSM1 was the top over-expressed gene in primary SCLC tissues compared to controls, increasing the reliability of the results. The potential of the DEGs identified in SCLC as diagnostic biomarkers was evaluated in an independent validation dataset (GSE40275), including transcriptomic data from primary SCLC and non-tumor lung tissues. The validation analysis verified 76% and 83.6% of the identified top 250 unique up-and down-regulated genes in SCLC, respectively, suggesting that these genes have the potential to serve as diagnostic biomarkers, prompting further validation.

Previous studies have reported human leukocyte antigen class II-based, chemotherapy- and Immune-related, m6A regulatory expression-based, cisplatin resistance-related risk signatures in SCLC [46,47,48,49,50,51,52]. These studies constructed risk signatures based on genes associated with a particular pathway or treatment response. Although this approach allows elucidating the importance of a pathway or mechanism in SCLC pathogenesis, it causes the number of genes screened for molecular signatures to be quite limited. A risk signature developed based on tumoral transcriptomic changes in SCLC patients has yet to be reported. In this study, based on the integrative transcriptome analysis results, 4394 differentially expressed genes identified in SCLC were considered candidates for the risk signature. Using Kaplan-Meier survival, Cox, and Lasso regression analyses, genes whose mRNA levels were not associated with prognosis were excluded by stepwise elimination, and the genes included in the signature were selected. Another difference in this study from previous studies was that only transcriptomic data from patients who had undergone curative-intent surgical resection were included in the analyses. Additionally, secondary tumors, biopsy samples, and patients with short follow-ups were not included in the study. While these sample exclusions contributed to reducing heterogeneity in patient cohorts, they also partially reduced the number of patients. It has been suggested that an EPV of 10 or more is needed to avoid the problem of overfitting in hazards regression analysis [53]. In this study, sample and event sizes were sufficient to conduct multivariate analysis for four predictors in the EGAD00001001244 cohort and two in GSE60052. This widely advocated rule of thumb for multivariate Cox regression analysis was followed in all analyses performed in this study. The parameters associated with survival outcomes of patients with resected SCLC vary between studies. Relevant factors that may indicate the prognosis of surgery for the clinical treatment of SCLC and the selection of candidates for surgery have previously been reported as smoking index, method of surgical resection, TNM stage of postoperative pathology, and postoperative chemotherapy [39,54]. In the Surveillance, Epidemiology, and End Results (SEER) Survey from 2010 to 2015, among the age, race, sex, stage, primary site, histologic type, chemotherapy and radiation parameters, only age, stage, and chemotherapy have been reported to be associated with survival in patients with resected SCLC [5]. Sex [55], visceral pleural invasion [56], lymphocyte-to-monocyte ratio [57], adjuvant chemotherapy after surgical resection [58], TNM stage and tumor location [59], and negative lymph node (NLN) [60] are among other factors reported to be associated with OS in patients who underwent surgical resection. In this study, sex and UICC stage were found to be associated with overall survival in the EGAD00001001244 and GSE60052 cohorts, respectively. Only the risk score was found to be associated with overall survival in both cohorts. As a result, a risk signature that can predict the overall survival of SCLC patients with higher performance than other clinical and demographic parameters was developed.

Expression values of 15 genes were used to construct the risk signature. These genes were ESRRG, GPT2, HTATSF1, PSRC1, RIMBP2, STRBP, SSX2IP, TRIM45, ZC3H8, EFNB2, EFHC2, PLLP, NR1H3, ZNF597, and CFAP126. ESRRG (estrogen-related receptor gamma) is a member of the estrogen receptor-related receptor family, and members of this family alter the expression of target genes by binding to response elements in DNA. In this study, the finding that ESRRG expression was higher in SCLC tumors than in the control group and that this higher expression was associated with increased overall survival suggested that ESRRG has the potential to act as a tumor suppressor in SCLC. Supporting data have also been previously reported in other types of cancer, such as colon cancer [61] and laryngeal squamous cell carcinoma [62]. HTATSF1 (HIV TAT Specific Factor 1) encodes a protein that functions as a transcription-splicing factor [63]. In non-small lung cancer, evidence has been reported that PGK1 regulates cancer metastasis by binding to HTATSF1 [64]. In this study, HTATSF1 clustered both with small nuclear ribonucleoproteins from the nucleocytoplasmic transport cluster in the up-regulated PPI network and with proteins from a cluster enriched in the ‘*antigen processing and presentation of exogenous peptide antigen* via *MHC Class II*’ category in the PPI network of down-regulated genes (Figure 12). However, the function of this protein in both cancer and eukaryotic cells is largely unknown and further studies are needed to elucidate its role in SCLC. It was interesting that five other genes involved in the risk signature (NR1H3, TRIM45, STRBP, ZNF597, and ZC3H8), along with ESRRG and HTATSF1, were also enriched in categories related to transcriptional regulation. ZC3H8 has been identified as a component of the LEC complex involved in the regulation of transcription of small nuclear RNAs (snRNA) by RNA polymerases II and III [65]. ZC3H8 expression has been reported to contribute to aggressive tumor cell behavior in breast cancer in vitro and in vivo models [66]. However, it has been associated with better survival in glioma patients [67]. In this study, ZC3H8 (Zinc Finger CCCH-Type Containing 8) was up-regulated in SCLC tissues, and patients with higher ZC3H8 expression tended to have worse overall survival. NR1H3 (Nuclear Receptor Subfamily 1 Group H Member 3) is a nuclear receptor and member of the NR1 family involved in macrophage function regulation and transcriptional regulation associated with lipid homeostasis and inflammation [68,69]. NR1H3 (Nuclear Receptor Subfamily 1 Group H Member 3) was down-regulated in SCLC, and its down-regulation was associated with decreased overall survival. These data were consistent with previous studies reporting NR1H3 as a tumor suppressor gene in different types of cancer [70,71,72,73]. TRIM45 (Tripartite Motif Containing 45) is an E3 ubiquitin-protein ligase that plays a role in regulating inflammatory response [74]. In this study, the mRNA expression of TRIM45 was higher in SCLC than in non-tumoral lung tissues, which correlated with increased overall survival. Similar results have been reported in brain tumors [75], non-small cell lung cancer [76], and breast cancer [77], underlining its tumor-suppressor function. It has been reported that ZNF597 (Zinc Finger Protein 597) may be a protective factor in osteosarcoma [78], but its role in cancer pathology remains unclear. In this study, expression of ZNF597 in SCLC was higher than in controls, and expression of this gene was associated with poor prognosis, indicating that ZNF597 may be a risk factor in SCLC. In this study, SCLC patients with low expression of RIMBP2 (RIMS Binding Protein 2), a protein predicted to be involved in neuromuscular synaptic transmission, had a poor prognosis, consistent with results previously obtained in lung squamous cell carcinoma patients [79]. EFNB2 (Ephrin B2), a receptor protein tyrosine kinase, had lower expression in SCLC than in control tissues, and low EFNB2 expression in patients was associated with decreased overall survival. It has been reported that Ephrin-B2 inhibits cell proliferation and motility in vitro and predicts longer metastasis-free survival in breast cancer [80]. In neuroblastoma, high expression of EFNB2 has been reported to predict favorable outcomes [81]. In breast cancer, EphB2 expression has been associated with a longer relapse-free survival [82]. There are also studies reporting a relationship between high EFNB2 expression and decreased survival [83], making it difficult to make definitive conclusions about the protein’s function in cancer pathology. In this study, proteins in the GPT2 and EFHC2 subnetworks were enriched in carbon and amino acid metabolism. GPT2 was up-regulated, and EFHC2 was down-regulated in SCLC tissues compared to non-tumoral lung tissues. Glutamic pyruvic transaminase 2 (GPT2), also known as alanine aminotransferase 2 (ALT2) expression, has been shown to be associated with good prognosis in hepatocellular carcinoma [84] and poor prognosis in breast cancer [85]. In this study, GPT2 mRNA expression was associated with a good prognosis. Further studies are necessary to clarify the clinical significance of GPT2 in SCLC. In this study, high expression of PLLP (Plasmolipin) was associated with poor prognosis in patients with SCLC. PLLP has been found to be up-regulated in brain tissues harboring metastases [86]; however, the clinical significance of PLLP in cancer has not yet been clarified. PSRC1 (Proline and Serine Rich Coiled-Coil 1) encodes a tumor suppressor protein p53- and DNA-damage down-regulated protein that plays an important role in mitosis and exhibits oncogenic characteristics [87]. In this study, PRSC1 high expression was associated with decreased overall survival. Similar results have been reported in hepatocellular carcinoma [88,89]. The PSRC1 sub-network was significantly enriched in the Cell Cycle category, suggesting that this gene may have a tumor-promoting effect in SCLC. 

The role of the identified prognostic genes in SCLC has not yet been reported. Therefore, to predict the pathways associated with the molecular pathogenesis of SCLC, GSEA, Gene Ontology, and PPI network analyses were first performed using the identified DEGs. Then the position of the pathways associated with the risk signature genes in the overall molecular pathogenesis was predicted. The pathways in which the identified differentially expressed genes are enriched were analyzed separately for up-regulated and down-regulated genes. The results of the GSEA and Gene Ontology analyses showed that the products of genes up-regulated in SCLC are primarily involved in cellular processes including mitosis, p53 signaling pathway, cell cycle, DNA repair, and transcriptional regulation. These pathways are well-known pathways that are closely related to cancer pathogenesis and are also the main targets of anti-cancer drugs [90,91,92]. The products of genes that were down-regulated in SCLC were found to be associated with the regulation of the immune response. 

In PPI analysis of up-regulated genes, proteins that interact closely with each other formed clusters enriched in Cell Cycle, DNA replication, DNA repair, CENP-A containing nucleosome assembly (containing proteins with roles in mitosis), and nucleocytoplasmic transport (containing proteins with roles in splicing of cellular pre-mRNAs) categories. The categories in which these clusters were enriched matched the results of some GSEA and GO analyses, including Cell Cycle, DNA Repair, mitosis, and transcriptional regulation, highlighting them as potential therapeutic targets in SCLC. Subsequent analyses revealed hub proteins involved in major pathways in SCLC pathogenesis, such as cell cycle control, DNA replication, and DNA repair. Interestingly, none of the identified hub proteins were associated with the overall survival of patients with SCLC who underwent surgical resection. However, the first neighbors of the proteins encoded by the genes in the defined risk signature included some hub proteins. At the same time, the functional analysis results of sub-PPI networks of genes belonging to the risk signature matched those of some protein clusters in the general PPI network. Notable among these were cell cycle and transcriptional regulation. In addition, the largest sub-PPI networks formed by genes included in the risk signature were enriched in the vasculature development category. Collectively, these results suggest that the pathways involved in overall survival in SCLC patients who have undergone surgical resection are cell cycle, transcriptional regulation, angiogenesis, and cellular metabolism. Indeed, these results are consistent with previously reported potentially treatable signaling pathways prominent in SCLC [93]. In this study, new prognostic potentially targetable proteins and related hub proteins involved in these pathways were reported. Further studies investigating the effectiveness of agents targeting identified prognostic genes or their interacting hub proteins may contribute to the development of new molecular treatment strategies.

It is also worth mentioning the down-regulated genes that are enriched in the immune response category. In the protein-protein interaction network analysis of the down-regulated genes, the pathways by which clustered gene products are involved were consistent with the GSEA and GO analysis results. It is known that tumor immunogenicity, determined by ‘antigenicity of tumor cells’ and ‘processing and presentation of tumor antigens’ [94], is weaker in SCLC than in other types of lung cancer [95,96]. The inflammatory state of the host immune system has been reported to be strongly associated with a good prognosis in patients with SCLC [97,98,99,100,101,102]. In addition, a high number of tumor-infiltrating lymphocytes have been associated with small tumor size, low tumor stage, and a favorable prognosis in operated SCLC [99]. Consistent with the results of previous studies [103,104], but in greater detail, this study demonstrated down-regulation of the genes encoding MHC Class I proteins, proteins involved in the associated IFN-I signaling pathway, and MHC Class II proteins (Figure 12 Cluster 1, 5, and Supplementary File S1A). CD8 + T and CD4+ lymphocytes recognize endogenous peptides presented by MHC Class I and exogenous peptides presented by MHC Class II proteins, respectively. It has been reported that SCLC can reduce MHC Class I expression through epigenetic programming [96,104], suggesting that there may be a mechanism to prevent CD8+ T cell-mediated rejection in SCLC. Although MHC Class I is expressed on almost all nucleated cells, MHC Class II is usually expressed by professional antigen-presenting cells (APCs). However, it is known that some cell types can express MHC Class II specifically by IFN-gamma. These include some NSCLC cell lines and tissues, and high MHC Class II expression has been associated with a good prognosis [105,106]. However, consistent with the results of this study, the absence of MHC Class II expression has been reported in SCLC [103], suggesting reduced presentation of tumor antigens to CD4+ T cells in SCLC. These results indicated that SCLC is deficient in antigen processing and presentation. Although some of the identified prognostic genes, including HTATSF1, NR1H3, and TRIM45, are known to have indirect immune-related functions, this study found no evidence of a direct correlation between overall survival and differential gene expression associated with reduced antigen processing and presentation in patients who underwent curative-intent surgical resection. Further evaluation of the predictive potential of the antigen processing and presentation pathway in larger patient cohorts is necessary to clarify its significance in patients with resected SCLC.

This study had limitations. First, this study was retrospective in nature and the results were based on analysis of publicly available datasets using bioinformatics. Second, the low sample size in SCLC, a known concern, remained a significant limitation for this study as well. Therefore, the results obtained need to be confirmed prospectively in larger patient cohorts. Additionally, the association between adjuvant chemotherapy in the EGAD00001001244 cohort and neoadjuvant chemotherapy in the GSE60052 cohort with the overall survival in patients could be evaluated. Although no significant association was found between chemotherapy and overall survival in this study, it can be speculated that chemotherapy may affect the model’s validity; therefore, this parameter should be considered in the validation of the model in further studies. Additionally, there are no reports on the functions of the proteins encoded by the identified prognostic genes in SCLC, and the majority of the genes still need to be adequately studied. In this study, the function of the identified prognostic genes in SCLC was predicted only by bioinformatics. Further functional studies are necessary to determine the role of these prognostic genes in SCLC. 

## 5. Conclusions

In this study, genes expressed differently in small cell lung cancer tumor tissues compared to non-tumoral lung tissues were identified using integrative transcriptome analysis. Then, for the first time in the literature, the mRNA levels of all genes found to be differentially expressed in SCLC were questioned for their potential relationships with the overall survival of patients. Thus, a robust multi-gene risk signature that could predict overall survival after curative-intent surgical resection in SCLC patients was constructed and validated. The power of the risk signature to discriminate between good and poor prognosis in patients was superior to existing clinical and demographic parameters. Therefore, the risk signature has the potential to serve preoperative clinical decision-making in predicting patient survival after curative surgical resection and deserves to be validated in a prospective study in larger patient groups. The results of this study also suggest that the most impacted pathways associated with survival after surgical resection in SCLC patients are primarily cell cycle, transcriptional regulation, angiogenesis, and cellular metabolism. Determining the functional importance of the identified prognostic genes that play roles in these pathways in SCLC pathogenesis may contribute to the development of new and more effective targeted treatment strategies.

## Figures and Tables

**Table 1:** Datasets used in the study.

Dataset ID	Platform	Tumor (*n*)	Control (*n*)	References
GSE149507	Affymetrix Human Genome U133 Plus 2.0 Array	18	18	Cai et al., 2021 [15]
GSE43346		23	-	Sato et al., 2013 [31]
GSE30219		21	14	Rousseaux et al., 2013 [26]
GSE40275	Human Exon 1.0 ST Array	5	14	Kastner et al., 2012 [27]
GSE108055	Illumina HumanWG-6 v2.0 expression beadchip	12	10	Asiedu et al., 2018 [28]
EGAD00001001244	Illumina HiSeq 2000	74	-	George et al., 2015 [21]
GSE60052		48	7	Jiang et al., 2016 [22]

**Table 2:** Clinical and demographic characteristics of the patients and the results of the univariate and multivariate Cox regression analyses.

				Univariate Analysis			Multivariate Analysis		
			*n*	*p*-Value	HR	(95% CI)	*p*-Value	HR	(95% CI)
**Training Cohort** **(*n* = 44)** **Event(*n*) = 23**	**Age**		44	0.21	1.03	(0.97–1.1)			
	**Sex**	Male	30	*Reference*					
		Female	14	0.26	0.57	(0.21–1.53)			
	**UICC Stage**	I + II	26	*Reference*					
		III + IV	18	**0.05**	2.29	(0.99–5.27)	0.12	1.99	(0.82–4.80)
	**Chemotherapy**	No	11	*Reference*					
		Yes	28	0.45	0.69	(0.27–1.79)			
	**Radiotherapy**	No	15	*Reference*					
		Yes	24	0.07	0.44	(0.18–1.07)			
	**Risk Score**		44	**<0.0001**	2.07	(1.67–2.55)	**<0.0001**	2.12	(1.59–2.82)
**Validation Cohort** **(*n* = 30)** **Event(*n*) = 23**	**Age**		44	0.68	0.99	(0.95–1.03)			
	**Sex**	Male	23	*Reference*					
		Female	7	**0.007**	0.06	(0–0.47)	**0.01**	0.08	(0–0.65)
	**UICC Stage**	I + II	19	*Reference*					
		III + IV	11	0.25	1.64	(0.70–3.83)			
	**Chemotherapy**	No	6	*Reference*					
		Yes	20	0.99	0.99	(0.32–3.03)			
	**Radiotherapy**	No	10	*Reference*					
		Yes	12	0.85	1.10	(0.36–3.32)			
	**Risk Score**		44	**0.002**	2.01	(1.28–3.15)	**0.04**	1.66	(1.02–2.69)
**Total Cohort** **(*n* = 74)** **Event(*n*) = 46**	**Age**		74	0.39	1.01	(0.98–1.05)			
	**Sex**	Male	53	*Reference*					
		Female	21	**0.007**	0.32	(0.14–0.73)	**0.01**	0.36	(0–0.82)
	**UICC Stage**	I + II	45	*Reference*					
		III + IV	29	0.38	1.36	(0.68–2.69)			
	**Chemotherapy**	No	17	*Reference*					
		Yes	48	0.70	0.87	(0.42–1.78)			
	**Radiotherapy**	No	25	*Reference*					
		Yes	36	0.13	0.60	(0.31–1.16)			
	**Risk Score**		74	**<0.0001**	2.07	(1.67–2.55)	**<0.0001**	2.04	(1.64–2.54)

## Data Availability

Data is available at NCBI GEO, accession numbers: GSE30219, GSE43346, GSE149507, GSE40275, GSE108055, GSE60052. Data (SCLC U Cologne Nature 2015/ EGAD00001001244) is available at both The European Genomephenome Archive (EGA) (https://ega-archive.org/datasets/EGAD00001001244 (accessed on 1 July 2022)) and The cBioPortal for Cancer Genomics (https://www.cbioportal.org/datasets (accessed on 1 July 2022)).

## Supplementary Materials

The following supporting information can be downloaded at: https://www.mdpi.com/article/10.3390/cancers15215219/s1, Supplementary File S1: (A) The identified DEGs in SCLC tissues compared to controls. The integrative-analysis revealed 1734 up-regulated and 2907 down-regulated unique genes in SCLC tissues compared to controls with an absolute difference bigger than 2-fold (FDR adj. *p* < 0.05). (B) A total of 173 genes were found to be related to overall survival in patients with SCLC (Cox-Mantel test, log-rank *p* < 0.05). (C) The result of the Cox regression analysis. (D) The identified hub proteins for the up-and down-regulated genes in SCLC.
